# Comparative Efficacy of Selenium Yeast Supplements on the Health and Productivity of Commercial Layers

**DOI:** 10.3390/ani16010023

**Published:** 2025-12-21

**Authors:** Muhammad Zain Ghauri, Muhammad Sharif, Ayesha Zafar, Umer Farooq, Muhammad Talha, Safdar Hassan, Usman Nazir, Dejun Ji

**Affiliations:** 1 College of Animal Science and Technology, Yangzhou University, Yangzhou 225009, China; 2 Institute of Animal and Dairy Sciences, University of Agriculture Faisalabad, Faisalabad 38000, Pakistan

**Keywords:** organic selenium, egg quality, immune response, commercial layers, nutrients digestibility

## Abstract

This study was conducted to evaluate the comparative efficacy of graded levels of dietary organic selenium (OS) on the health, productivity, and egg quality of commercial laying hens. A total of 240 hens (26 weeks old) were randomly allotted to four dietary treatments in a completely randomized design: a control basal diet (0 ppm OS) and three diets supplemented with OS from selenium yeast (SY-2000 or SY-3000) to achieve levels of 0.20, 0.30, and 0.45 ppm, respectively. The experiment lasted for 72 days. Performance metrics were recorded weekly, and samples were collected at two time points to assess physiological responses. The results demonstrated that supplementation with 0.30 and 0.45 ppm OS significantly improved key productivity parameters. Egg production (*p* < 0.05), egg weight (*p* < 0.001), and cumulative egg mass (*p* < 0.001) were higher in these groups compared to the control. Nutrient digestibility, including dry matter, crude protein, and ether extract, was also significantly enhanced (*p* < 0.001) at the higher OS levels. The antioxidant defense system was strengthened, as evidenced by the increased serum activity of the glutathione peroxidase (*p* < 0.001), superoxide dismutase (*p* < 0.01), and diphenyl picrylhydrazyl radical scavenging capacity (*p* < 0.05). Furthermore, the humoral immune response against Newcastle disease showed a significant, dose-dependent improvement (*p* < 0.001). In contrast, feed intake, various egg quality parameters (shell thickness, specific gravity, and Haugh unit), and serum mineral profiles remained unaffected (*p* > 0.05) by OS supplementation. In conclusion, dietary OS supplementation at 0.30–0.45 ppm effectively enhances laying performance, nutrient utilization, systemic antioxidant capacity, and specific immunity, with the 0.45 ppm level demonstrating the most consistent and comprehensive benefits under the conditions of this study.

## Simple Summary

Organic selenium supplementation is crucial for enhancing the productivity and health of commercial laying hens. This study highlights its specific role in improving key performance metrics. As a vital micronutrient, organic selenium significantly boosts nutrient digestibility and egg quality parameters. Furthermore, it plays an essential role in reinforcing the hens’ antioxidant defense systems and enhancing immune responses, particularly against challenges like Newcastle disease. The findings confirm that dietary organic selenium is a fundamental nutritional strategy for optimizing layer welfare and sustainable egg production, moving beyond conventional performance outcomes to support overall physiological resilience.

## 1. Introduction

The productivity of the poultry industry has been enhanced in recent decades due to the consistent improvement in genetics. In correspondence to these improvements, nutritional requirements must be frequently redefined according to local economic, environmental and managemental conditions [1]. Minerals are the inorganic portion of the feedstuff, essential for performing different physiological and biological activities in poultry. The body needs micro minerals (trace elements) in a small quantity such as chromium, copper, fluorine, iodine, iron, manganese, molybdenum, zinc, and selenium [2]. Trace elements play an important role in the biological functioning of poultry, including development, reproduction, and antioxidant defense [3]. Selenium as an essential element plays an important biological role in the animal body [4]. It is the essential component of an enzyme known as glutathione peroxidase (*GSH-Px*) that performs a fundamental role in antioxidation [5].

The levels of Se present in the tissues of plants are directly related to the amount present in the soil. Generally, its distribution around the globe is not uniform, which influences the concentration changes of Se present in the tissues of the birds and human food [5,6]. The heterogeneity of Se with the supplementation of organic selenium (OS) in the diets of birds resulted in better growth and immunity. Today, poultry production practices bring with them stress factors and intense metabolism in birds, often leading to an increase in free radicals’ production. The result obtained from the experiment showed decreased bird performance by increased health problems, leading to low-quality end products. Selenium prevents birds from experiencing stress and acts as an antioxidant because SY has seleno-methionine (SeMet) with the addition of a little quantity of Se from other substances [7,8].

In the poultry feed industry, selenium-enriched yeast (*SY*) and sodium selenite (*SS*) are primarily used as organic and inorganic Se, respectively [5,6]. The influence of various Se levels on poultry production has become the subject of many studies. Generally, it is known that the Se from sodium selenite (*SS*) is more toxic than Se from SY because the maximum tolerate level (0.2 to 0.3 mg/kg) of SY is much higher compared to the tolerate level of Se from SS in birds [9,10]. Organic Se is gaining more attention due to the lower toxicity of SY [11]. The bioavailability of SY is more than SS but SY is less toxic than SS. It is reported that the bioavailability of OS such as SY is greater than that of inorganic selenium such as SS for the tissue concentration [5,12]. The supplementation of Se in animals’ diet affects the oxidative processes and physiological changes in birds. The SY was more effective in increasing tissue enrichment, increasing the immunity and antioxidative ability in animals [4,13]. Ref. [1] also observed that the dietary supplementation of OS in laying hens had increased the serum peroxidase in laying hens to protect them against free radical and carcinogenic factors with its antioxidant properties. Selenium is beneficial for immunity and growth, so diets deficient in Se are supplemented for better results. The use of SS in diet resulted in increased FI [6].

OS boosted growth and reduced the impact of stress in commercial farming, leading to low mortality rates and improved flock uniformity. Methionine, heavy metals, and vitamin C are the main dietary factors affecting Se utilization [14,15]. Se in the poultry diet had shown nutritional benefits for the consumers who ate Se-rich poultry products. Selenium yeast had shown an antiviral effect, which reduced the risk of autoimmune thyroid disease, and helped to prevent cancer [1,3,4]. The study regarding the effects of the supplementation of organic Se on egg quality, production, and immune responses in layers is still needed. Therefore, the main goal of this trial is to determine the effectiveness of organic Se in production, egg quality, and immune response in layers.

## 2. Materials and Methods

### 2.1. Ethical Statement

All experimental procedures and animal care protocols were approved by the Advanced Studies and Research Board, University of Agriculture, Faisalabad (DGS/2021/3163) and the Institutional Animal Care and Use Committee of Yangzhou University (License No. SYXK(Su)2021-0027).

### 2.2. Experimental Design and Management

Prior to the experiment, the hens’ house and equipment were thoroughly sanitized through high-pressure washing and disinfection, followed by ventilation and air purification, to ensure a pathogen-controlled environment. The experimental trial was executed at Raja Muhammad Akram, Animal Nutrition Research Center, University of Agriculture, Faisalabad. A total of 240 white commercial layers (Crystal- Nick purchased from Asia chicks pvt Ltd-Pakistan, Lahore, Pakistan) at 26 weeks of age were randomly allocated into 4 treatment groups, with 5 replicates per group and 12 birds per replicate. The trial lasted 72 days. The experimental birds were reared in single-tier cage system. Light bulbs were installed to provide light for 17 h. Temperature, relative humidity, and ammonia level were regulated according to the bird’s requirements.

### 2.3. Diet Formulation

Four diets were prepared and these diets were supplemented with 0, 100, and 150 g/ton SY-2000 and 150 g/ton SY-3000, containing 0, 0.20, 0.30, and 0.45 ppm OS, and were named as OS 0, OS 0.20, OS 0.30, and OS 0.45, respectively. SY-2000 was purchased from Ghazi Brother Pvt. Ltd. Under the brand name of Selemax^®^, it consists of a specific strain of inactivated Saccharomyces cerevisiae yeast (NCYC R646) that has been enriched with organic selenium compounds, primarily seleno-methionine, during the fermentation process. It is available in concentrations of 1000 ppm and 2000 ppm (we used Selemax 2000; Ghazi Brothers Pvt Ltd, Karachi, Pakistan).

While SY-3000 was purchased from Angel Yeast Co. Ltd. (Yichang, China) under the brand name of Yeasel^®^, it was produced by submerged Saccharomyces cerevisiae on a selenium-enriched media. Its guaranteed analysis shows 98% purity and >2000 or 3000 ppm selenium. The formulation of the experimental diet was primarily based on the nutrient requirements for laying hens recommended by the Nutrient Requirements of Poultry and Crystal- Nick feed manual. The basal diet was prepared using corn, soybean meal, and wheat bran as primary ingredients. The contents of crude protein, crude fiber, methionine, and lysine in these basal ingredients were determined via near-infrared spectroscopy (*NIRS*). The detailed composition and nutritional profile of the diet are presented in Table 1.

### 2.4. Parameter Analysis

Feed intake, egg production, egg weight, and egg mass were documented. Moisture, crude fat, crude protein, crude fiber, acidity, and ash were carried out by proximate analysis (proposed by AOAC in 2000). Serum minerals were evaluated by using technique of flame photometer for Na and K, and titration method for Ca, Mg, Cl, and P by using spectrophotometer [16]. These procedures were all carried out after wet digestion proposed by AOAC in 2000. At the end of the trial, blood samples were collected from the brachial vein of hens at 35 wk of age. After collection, the samples were allowed to clot at room temperature for 30 min. Subsequently, they were centrifuged at 3000 rpm for 10 min at 4 °C to separate serum. The serum was aliquoted and stored at −20 °C for further biochemical analyses. Antioxidant status was measured using kits (Glutathione peroxidase (GSH-px); A005-1-2, Superoxide Dismutase (SOD); A001-3-2 & Diphenyl picrylhydrazyl (DPPH); A153-1-1), purchased from Nanjing Jiancheng Bioengineering Co., Ltd. (Nanjing, China). In this trial, eggs were collected for evaluation at 36th and 72nd days of the trial. Two eggs from each replicate were collected for evaluation. Standard practices usually involved selecting uncracked eggs. Specific gravity was determined using the flotation method involving different salt concentrations in water. The specific gravity was calculated using various salt concentrations (276, 298, 320, 342, 365, 390, 414, 438, and 462 g) per three liters of water. The egg weight, albumen height, Haugh unit, and egg yolk color were measured using an egg analyzer (EMT-7300, Robotmation Co., Ltd., Tokyo, Japan). Egg shape index = transverse diameter/longitudinal diameter, eggshell ratio (%) = eggshell weight x 100/whole egg weight; egg yolk ratio (%) = weight of egg yolk × 100/weight of whole egg; the shell strength was measured using a shell strength instrument (AC220, Orka Company, Shanghai, China); the shell thickness (remove the inner shell membrane) was measured at three locations (equator, blunt, and sharp ends), and the values recorded at the 3 locations were averaged. The shell thickness was measured using an eggshell thickness gauge (Guilin Measuring Tool Cutting Tool Co., Ltd., Guilin, China). The hemagglutinin inhibition test was used in which titer against NDV was tested using HPLC by following [17]’s study. Sixteen hemagglutinin units of the ND antigen were used to check the antibody titers against NDV. Digestibility trial was conducted to estimate nutrient digestibility (dry matter, crude protein, and crude fiber) by indirect marker method using acid insoluble ash. Experimental diet was supplemented with 1% Celite^®^ (Lompoc, CA, USA) last three days of trials. On 70th, 71st, and 72nd day of trial, bird droppings were collected. Droppings were collected twice a day and were spread over polythene sheet. Bird droppings were collected from individual replicate after 24 h of feeding. Foreign particles and feathers were removed carefully from droppings. Droppings were collected in 3 days from each replicate as composite sample and was weighed, mixed, oven-dried at 65 °C, and ground for analysis, and acid insoluble ash in feed and droppings were determined. Determination of acid insoluble ash (AIA) was performed after ashing the samples and treating the ash with boiling HCl [18]. The digestibility was determined by the following formula:

Digestibility coefficient (%) = 100 − [100 × (AIA in diet %/AIA in feces%) × (nutrients in feces%/nutrients in the diet%)]

Weekly egg production percentage was calculated on a hen-day basis using the following formula:

Egg production (%) = (Total eggs collected in the week/(Average number of hens alive in the week × 7)) × 100.

### 2.5. Statistical Analysis

Experimental data were initially organized using Microsoft Excel 2019. All statistical analyses were performed using SPSS 21.0 (IBM Corp., New York, NY, USA). The effect of Se concentration was analyzed at each sampling time point using a one-way analysis of variance (ANOVA). Where the ANOVA indicated a significant effect (*p* < 0.05) at a given time point, differences among specific treatment groups were identified for that time point using Duncan’s multiple range test. Results are presented as means ± the pooled standard error of the mean (SEM).

## 3. Results

### 3.1. Effect of Organic Selenium on Feed Intake of Laying Hens

Table 2 shows the effect of the dietary supplementation of different levels of OS (OS 0, OS 0.20, OS 0.30, and OS 0.45) on FI in laying hens from week 26th to 35th. A statistical analysis showed a non-significant difference (*p >* 0.05) between the treatments on FI in laying hens throughout the trial.

### 3.2. Effect of Organic Selenium on Egg Production of Laying Hens

The data from this study showed that the egg production in the first week of the experimental trial was 88.574, 90.48, 91.432, and 93.336% with OS 0, OS 0.20, OS 0.30, and OS 0.45, respectively. The higher value of egg production was shown by OS 0.45 as compared to other treatments. Similar results were found in 2nd, 3rd, 4th, 5th, 6th, 7th, 8th, 9th, and 10th weeks of the trial (Table 3).

### 3.3. Effect of Organic Selenium on Egg Mass of Laying Hens

Similar results to those of egg production were found on the weekly egg mass by different dietary treatments (Table 4). The OS 0.45 showed a significantly increased egg mass by organic selenium supplementation in the laying hens’ diet throughout the research trial.

### 3.4. Effect of Organic Selenium on Egg Weight of Laying Hens

Once more, OS 0.45 showed the maximum egg weight as compared to the other treatment groups during all weeks of the experiment (Table 5).

### 3.5. Effect of Organic Selenium on Egg Quality of Laying Hens

Table 6 has been presenting the results regarding the egg quality parameters at different intervals. All these parameters like eggshell thickness, specific gravity, egg length, and Haugh unit score were kept constant by all groups at both intervals.

### 3.6. Effect of Organic Selenium on Nutrient Digestibility of Laying Hens

The results of the dietary supplementation of different levels of OS on the nutrient digestibility in laying hens at the end of the trial are represented in Table 7. The maximum (*p <* 0.05) digestibility of DM was observed in the OS 0.30- and OS 0.45-supplemented diets while the minimum (*p* < 0.05) digestibility of DM was observed in the OS 0 diet in laying hens. Likewise, the maximum CP and EE digestibility were observed in the OS 0.45-supplemented diet, while the minimum digestibility of CP was observed in the OS 0 diet in laying hens.

### 3.7. Effect of Organic Selenium in the Diet of Laying Hens on Antioxidation Activity

Table 8 shows the results of the dietary supplementation of different levels of OS on antioxidant activity in laying hens on the 36th and 72nd days of the trial. The GSH-px concentration was the maximum in the OS 0.3 group on both days, while the activity of SOD and DPPH were the highest in the 0.45 ppm supplementation group.

### 3.8. Effect of Organic Selenium in the Diet of Laying Hens on Serum Minerals

The effects of organic selenium supplementation on serum mineral concentrations are presented in Table 9. A statistical analysis showed a non-significant effect (*p > 0.05*) between the treatments on the serum mineral (Ca, P, Mg, K, Cl, and Na) concentration in laying hens.

### 3.9. Effect of Organic Selenium in the Diet of Laying Hens’ Bone Mineralization

Table 10 shows the effect of the dietary supplementation of different levels of OS on the bone mineralization in laying hens on the 36th and 72nd days of the trial. The results of tibia ash, Ca, and P were observed to be non-significant (*p >* 0.05) in response to the addition of OS in the diet of layer.

### 3.10. Effect of Organic Selenium in the Diet of Laying Hens on Antibody Titer Against Newcastle Disease

The results of the present study showed that the effects of the different levels of organic Se on antibody titers against ND in laying hens on day 36th and 72nd of the trial were significant (*p <* 0.05) as shown in Table 11. The data from this study showed that antibody titers against ND on the 36th day of the experimental trial were 5.081, 6.056, 6.404, and 6.917 with OS 0, OS 0.20, OS 0.30, and OS 0.45, respectively. The higher value of the antibody titer against ND was shown by T4, compared to the other treatments. Antibody titers against ND on the 72nd day of the experimental trial were 6.488, 9.201, 9.549, and 9.829 with OS 0, OS 0.20, OS 0.30, and OS 0.45, respectively. The higher value of the antibody titer against ND was shown by T4, compared to the other treatments.

## 4. Discussion

The present study provides a comprehensive evaluation of the effects of supplementing the diets of commercial laying hens with varying levels of organic selenium in the form of selenium yeast, from a baseline of 0 ppm up to 0.45 ppm. The results unequivocally demonstrate that selenium supplementation, particularly at the higher levels of 0.30 and 0.45 ppm, confers significant benefits on key productivity parameters, nutrient utilization, antioxidant status, and immune function, without exerting any adverse effects on feed intake, egg quality, or serum mineral homeostasis. This detailed discussion will interpret these findings in the context of the physiological roles of selenium and the existing body of scientific literature, elucidating the mechanisms behind the observed effects and their practical implications for the poultry industry [19].

One of the most fundamental and reassuring findings of this trial was the lack of any significant effect of organic selenium supplementation on feed intake across all treatment groups. This is a critical observation from both a physiological and an economic standpoint. It indicates that the inclusion of selenium yeast, even at the highest level of 0.45 ppm, does not impart any palatability issues or negatively affect the hens’ voluntary consumption of feed [20]. This finding aligns with numerous previous studies that reported no change in FI in the layers or broilers fed organic selenium [21,22,23]. The consistency in FI across treatments is crucial because it establishes that the subsequent improvements in production performance are not due to increased nutrient consumption but rather to enhanced internal physiological processes mediated by selenium. It allows us to isolate the effects of selenium on the metabolism and the utilization of a constant nutrient input, rather than confounding it with variations in intake [1,3,4].

The most pronounced and consistent benefits of organic selenium supplementation were observed in the core productivity metrics: egg production, egg weight, and, consequently, egg mass [24,25]. The results demonstrated a clear, dose-dependent response, with the OS 0.45 group consistently outperforming the control (OS 0) and lower-level supplementation groups throughout the 10-week trial. The superiority of the OS 0.45 treatment was statistically significant from the very first week, and it maintained this advantage, culminating in a remarkable 98.57% egg production rate by week 10, compared to 95.95% in the control group. Similarly, the egg weight and egg mass showed a steady, significant increase in the selenium-supplemented groups, with the highest values consistently recorded for the OS 0.45 treatment [26]. These results are in strong agreement with a substantial body of research. The underlying mechanism for this enhanced productivity is multifaceted but is primarily attributed to selenium’s role as an integral component of the antioxidant defense system [21]. Selenium is a crucial constituent of the enzyme glutathione peroxidase, which is responsible for neutralizing hydrogen peroxide and lipid hydroperoxides within cells. In the highly metabolically active environment of the laying hen, particularly in the liver and the ovarian tissue, rampant lipid metabolism and steroidogenesis generate significant oxidative stress [27]. By bolstering the GPx system, organic selenium protects the cellular integrity of hepatocytes and follicular cells from oxidative damage. This preservation of cellular function ensures the optimal synthesis of yolk precursors (such as vitellogenin) in the liver and supports the maturation and ovulation of follicles, thereby directly translating into higher and more sustained rates of egg production and improved yolk development, which contributes to the overall egg weight [28].

This proposed mechanism is strongly corroborated by our findings on antioxidant activity. The data on serum antioxidant enzymes peroxidase, *SOD*, and *DPPH* radical scavenging activity provide direct biochemical evidence for the enhanced antioxidant capacity conferred by selenium yeast [29,30]. The significantly higher activities of these enzymes in the OS 0.30 and OS 0.45 groups, especially by the 72nd day, confirm a systemic upregulation of the birds’ endogenous defense systems. SOD serves as the first line of defense, catalyzing the dismutation of the superoxide radical into oxygen and hydrogen peroxide, which is then efficiently decomposed by GPx and other peroxidases [4,31]. The elevated DPPH scavenging activity further indicates a general enhancement in the serum’s non-enzymatic antioxidant potential. A reduction in oxidative stress within the gastrointestinal tract could also explain the observed improvements in nutrient digestibility [8]. Our results clearly show that supplementation with 0.30 and 0.45 ppm organic selenium significantly increased the digestibility of DM, CP, and *EE*. The intestinal mucosa is highly susceptible to oxidative damage from dietary peroxides and endogenous metabolic by-products. This damage can compromise the integrity and function of enterocytes, leading to reduced digestive enzyme activity and impaired nutrient absorption. By integrating into seleno-proteins within the gut mucosa, organic selenium protects these cells, thereby maintaining optimal digestive and absorptive function [1,32]. The improved EE digestibility is particularly noteworthy, as fats are highly prone to peroxidation, and their efficient digestion and absorption are critical for providing energy and lipid components for yolk formation. The enhanced CP digestibility similarly ensures a better supply of amino acids for albumen synthesis and overall body maintenance. Therefore, the synergistic effect of reduced oxidative stress on both the reproductive system and the digestive tract creates a powerful driver for improved productivity: better nutrient absorption from the same amount of feed, coupled with the more efficient utilization of these nutrients for egg synthesis [14].

A particularly compelling finding of this study is the significant enhancement in the humoral immune response, as measured by antibody titers against Newcastle disease (*ND*). The antibody titers showed a clear dose–response relationship, with the OS 0.45 group achieving the highest values on both the 36th and 72nd days. This immunomodulatory effect of selenium is well-documented and is again rooted in its antioxidant function [33,34]. Lymphocytes, the cells responsible for antibody production, are exceptionally sensitive to oxidative stress. Reactive oxygen species can impair lymphocyte proliferation, differentiation, and function. By mitigating oxidative stress, selenium helps maintain a robust and responsive immune system. Furthermore, selenium is known to influence the expression of cytokines and other signaling molecules that orchestrate the immune response [35]. An enhanced antibody titer indicates a more effective vaccination response and the better preparedness of the bird to combat infectious challenges, which is a critical aspect of flock health and welfare in intensive production systems [25]. This finding moves the benefits of selenium supplementation beyond mere productivity, positioning it as a valuable tool for enhancing overall flock resilience and reducing the reliance on therapeutic antibiotics [23,36].

In contrast to the significant effects on production, digestion, and immunity, the results for egg quality parameters (shell thickness, specific gravity, egg length, and Haugh unit) and serum mineral levels (Ca, P, Mg, K, Cl, and Na) revealed no significant differences among the treatments. The stability of the egg quality parameters is interesting. While some studies have reported improvements in the shell quality due to selenium’s role in the antioxidant protection of the shell gland mucosa [37], our results suggest that, under the conditions of this trial, the basal diet and the health status of the hens were sufficient for maintaining shell integrity and internal egg quality [24,38,39]. The non-significant findings for serum minerals indicate that selenium supplementation, within the tested range, does not disrupt the homeostatic regulation of these major electrolytes and minerals. This is a positive outcome, confirming the safety of the supplementation regimen [22,26]. Similarly, the parameters of bone mineralization (tibia ash, Ca, and P) showed no statistically significant improvements, although a clear trend towards increased values in the OS 0.30 and OS 0.45 groups was observable, with *p*-values approaching significance (0.054 to 0.079). This suggests that, while selenium may have a positive influence on bone health, perhaps by protecting bone-forming cells from oxidative stress, the effect might be more subtle or require a longer duration to become statistically definitive compared to the more direct and rapid effects on the liver and oviduct [22,26,28,40,41].

The study demonstrates a clear dose–response efficacy hierarchy. While the 0.20 ppm OS level showed improvements over the control, the 0.30 and 0.45 ppm doses yielded the most consistent and significant benefits in the production parameters and nutrient digestibility. Concerning the oxidative status, these higher doses, particularly 0.45 ppm, significantly elevated the serum SOD activity and DPPH radical-scavenging capacity [29]. However, interpreting these elevated enzymatic activities requires caution, as they may indicate either an enhanced antioxidant defense or a compensatory response to an increased oxidative challenge [42]. In the absence of concomitant markers of oxidative damage, we conclude that supplementation primarily altered the antioxidant status. Crucially, these changes occurred alongside improved immune indices and production performance without adverse effects, suggesting an overall benefit [43]. The 0.45 ppm level, being within the EU regulatory limit of 0.5 ppm for poultry feed, represents a safe and effective strategy to leverage selenium’s bioactivity, potentially offering a marginal advantage in modulating the systemic antioxidant capacity and supporting productivity.

## 5. Conclusions

In conclusion, the dietary supplementation of organic selenium (Se-yeast) in laying hens elicited a clear, dose-dependent response, with 0.45 ppm emerging as the most effective level for enhancing productivity and health. The primary benefits were observed in the performance metrics: the 0.45 ppm supplementation significantly improved the egg production, egg weight, and egg mass throughout the 10-week trial without affecting the feed intake. These performance gains were supported by enhanced nutrient digestibility (dry matter, crude protein, and ether extract) and a marked improvement in the systemic antioxidant capacity, as shown by the increased serum peroxidase, superoxide dismutase, and radical scavenging activity. Furthermore, organic selenium potentiated the humoral immune response, yielding significantly higher antibody titers against Newcastle disease. Importantly, the supplementation did not significantly alter several other measured parameters. The egg quality (shell thickness, specific gravity, egg length, and Haugh unit), serum mineral profiles (Ca, P, Mg, K, Cl, and Na), and tibia bone mineralization metrics remained unaffected by dietary selenium levels within the studied range.

Therefore, organic selenium at 0.45 ppm acts as a functional nutrient that enhances productivity primarily by improving metabolic efficiency and mitigating oxidative stress, thereby supporting immune competence. Its incorporation into layer diets is recommended in order to bolster overall flock performance and health, while it is understood that its primary mode of action does not extend to altering the egg quality or mineral homeostasis under the conditions of this study.

## Figures and Tables

**Table 1:** Composition and nutrient levels of basal diets (air-dry basis).

Ingredients	Treatments (%)
Corn	60.5
Soybean meal	29.0
Wheat bran	6.8
Limestone	1.0
CaHPO_4_	1.3
DL-Met	0.1
NaCl	0.3
Premix	1.0
Total	100.0
Nutrient levels	
ME/(MJ/kg)	11.49
CP	18.75
CF	3.32
Ca	0.80
TP	0.63
AP	0.32
Met	0.40
Lys	0.97

The premix was provided by the “YangDa” Feed Company (Yangzhou, China). One kilogram of premix contained the following: vitamin A, 1,250,000 IU; vitamin B_1_, 200 mg; vitamin B_2_, 750 mg; vitamin B_6_, 375 mg; vitamin B_12_, 2.25 mg; vitamin D_3_, 375,000 IU; vitamin E, 2500 mg; vitamin K_3_, 200 mg; *D*-biotin, 15 mg; folic acid, 125 mg; *D*-pantothenic acid, 1125 mg; nicotinamide, 3250 mg; choline, 50 g; phytase, 300,000 U; Cu (as copper sulfate), 0.95 g; Fe (as ferrous sulfate), 9.5 g; Mn (as manganese sulfate), 9.5 g; Zn (as zinc sulfate), 9 g; I(as calcium iodate), 57.5 mg; and Se (as sodium selenite), 40 mg.

**Table 2:** Effect of organic selenium in the diet of laying hens on feed intake.

Week	Feed Intake (g)				SEM	*p*-Value
	OS 0	OS 0.20	OS 0.30	OS 0.45		
**Week 26**	739	748	754	770	15.651	0.317
**Week 27**	731	740	740	740	4.702	0.418
**Week 28**	735	734	734	735	0.721	0.570
**Week 29**	770	770	770	770	0.690	0.418
**Week 30**	767	770	770	767	2.201	0.581
**Week 31**	725	743	761	745	25.042	0.787
**Week 32**	768	770	769	770	0.913	0.651
**Week 33**	770	769	770	770	0.401	0.121
**Week 34**	770	770	769	770	0.503	0.418
**Week 35**	770	770	770	768	1.001	0.418

OS 0, OS 0.20, OS 0.30, and OS 0.45 represent supplementation of 0, 0.20, 0.30, and 0.45 ppm OS in basal diet; SEM: standard error of means.

**Table 3:** Effect of organic selenium on egg production on weekly basis.

Week	Egg Production (%)				SEM	*p* Value
	OS 0	OS 0.20	OS 0.30	OS 0.45		
Week 1	88.574 ^c^	90.480 ^b^	91.432 ^b^	93.336 ^a^	0.446	0.001
Week 2	90.480 ^c^	91.670 ^bc^	92.860 ^b^	94.288 ^a^	0.347	0.001
Week 3	91.670 ^b^	92.860 ^b^	94.288 ^a^	94.764 ^a^	0.326	0.001
Week 4	92.860 ^c^	94.288 ^b^	95.240 ^ab^	95.716 ^a^	0.326	0.001
Week 5	94.288 ^b^	95.240 ^ab^	95.954 ^a^	96.430 ^a^	0.326	0.002
Week 6	95.240 ^b^	95.954 ^ab^	96.430 ^ab^	96.906 ^a^	0.337	0.019
Week 7	95.954 ^a^	96.430 ^a^	96.430 ^a^	96.906 ^a^	0.337	0.029
Week 8	95.954 ^a^	96.430 ^a^	96.430 ^a^	97.144 ^a^	0.337	0.137
Week 9	95.954 ^b^	96.430 ^b^	96.430 ^b^	97.858 ^a^	0.326	0.005
Week 10	95.954 ^b^	96.430 ^b^	96.430 ^b^	98.572 ^a^	0.376	0.001

OS 0, OS 0.20, OS 0.30, and OS 0.45 represent supplementation of 0, 0.20, 0.30, and 0.45 ppm OS in basal diet; SEM: standard error of means. Values labeled with different lowercase letter within a column indicate significant differences between groups (*p* < 0.05).

**Table 4:** Effect of organic selenium on egg mass on weekly basis.

Week	Egg Mass (g)				SEM	*p* Value
	OS 0	OS 0.20	OS 0.30	OS 0.45		
Week 1	51.614 ^d^	52.768 ^c^	53.888 ^b^	54.696 ^a^	0.064	0.001
Week 2	52.768 ^d^	53.888 ^c^	54.696 ^b^	55.516 ^a^	0.073	0.001
Week 3	53.888 ^d^	54.696 ^c^	55.516 ^b^	56.208 ^a^	0.078	0.022
Week 4	54.696 ^d^	55.516 ^c^	56.208 ^b^	56.644 ^a^	0.084	0.000
Week 5	55.516 ^d^	56.208 ^c^	56.644 ^b^	57.096 ^a^	0.083	0.005
Week 6	56.208 ^d^	56.644 ^c^	57.096 ^b^	57.528 ^a^	0.067	0.001
Week 7	56.644 ^d^	57.096 ^c^	57.528 ^b^	57.864 ^a^	0.067	0.030
Week 8	57.096 ^d^	57.528 ^c^	57.864 ^b^	58.284 ^a^	0.065	0.007
Week 9	57.528 ^d^	57.864 ^c^	58.284 ^b^	58.656 ^a^	0.054	0.012
Week 10	57.864 ^d^	58.284 ^c^	58.656 ^b^	58.920 ^a^	0.055	0.033

OS 0, OS 0.20, OS 0.30, and OS 0.45 represent supplementation of 0, 0.20, 0.30, and 0.45 ppm OS in basal diet; SEM: standard error of means. Values labeled with different lowercase letter within a column indicate significant differences between groups (*p* < 0.05).

**Table 5:** Effect of organic selenium on egg weight on weekly basis.

Week	Egg Weight (g)				SEM	*p* Value
	OS 0	OS 0.20	OS 0.30	OS 0.45		
Week 1	51.614 ^d^	52.768 ^c^	53.888 ^b^	54.696 ^a^	0.064	0.007
Week 2	52.768 ^d^	53.888 ^c^	54.696 ^b^	55.516 ^a^	0.073	0.020
Week 3	53.888 ^d^	54.696 ^c^	55.516 ^b^	56.208 ^a^	0.078	0.011
Week 4	54.696 ^d^	55.516 ^c^	56.208 ^b^	56.644 ^a^	0.084	0.004
Week 5	55.516 ^d^	56.208 ^c^	56.644 ^b^	57.096 ^a^	0.083	0.001
Week 6	56.208 ^d^	56.644 ^c^	57.096 ^b^	57.528 ^a^	0.067	0.038
Week 7	56.644 ^d^	57.096 ^c^	57.528 ^b^	57.864 ^a^	0.067	0.021
Week 8	57.096 ^d^	57.528 ^c^	57.864 ^b^	58.284 ^a^	0.065	0.030
Week 9	57.528 ^d^	57.864 ^c^	58.284 ^b^	58.656 ^a^	0.054	0.011
Week 10	57.864 ^d^	58.284 ^c^	58.656 ^b^	58.920 ^a^	0.055	0.041

OS 0, OS 0.20, OS 0.30, and OS 0.45 represent supplementation of 0, 0.20, 0.30, and 0.45 ppm OS in basal diet; SEM: standard error of means. Values labeled with different lowercase letter within a column indicate significant differences between groups (*p* < 0.05).

**Table 6:** Effect of organic selenium on egg quality parameters at different time intervals.

Egg quality	Treatments				SEM	*p* Value
	OS 0	OS 0.20	OS 0.30	OS 0.45		
**At 36th day**						
Specific gravity	1.080	1.088	1.091	1.080	0.050	0.750
Shell thickness (mm)	39.900	40.100	40.000	41.000	0.473	0.360
Egg length (mm)	60.247	59.329	60.356	60.519	0.518	0.392
Haugh unit score	83.457	88.629	84.895	86.554	1.338	0.076
**At 72th day**						
Specific gravity	1.082	1.074	1.076	1.079	0.065	0.313
Shell thickness (mm)	40.550	39.200	38.400	39.000	0.731	0.243
Egg length (mm)	57.528	56.846	58.233	57.754	0.699	0.577
Haugh unit score	83.821	88.291	83.639	86.264	1.913	0.298

OS 0, OS 0.20, OS 0.30, and OS 0.45 represent supplementation of 0, 0.20, 0.30, and 0.45 ppm OS in basal diet; SEM: standard error of means.

**Table 7:** Effect of organic selenium in the diet of laying hens on nutrient digestibility.

Digestibility (%)	Treatments				SEM	*p*-Value
	OS 0	OS 0.20	OS 0.30	OS 0.45		
**Dry matter (%)**	77.37 ^b^	77.52 ^b^	78.43 ^a^	78.43 ^a^	0.040	0.031
**Crude protein (%)**	71.39 ^b^	70.54 ^c^	71.73 ^a^	71.78 ^a^	0.050	0.015
**Ether extract (%)**	77.74 ^d^	78.85 ^b^	78.63 ^c^	79.51 ^a^	0.032	0.029

OS 0, OS 0.20, OS 0.30, and OS 0.45 represent supplementation of 0, 0.20, 0.30, and 0.45 ppm OS in basal diet; SEM: standard error of means. Values labeled with different lowercase letter within a column indicate significant differences between groups (*p* < 0.05).

**Table 8:** Effect of organic selenium in the diet of laying hens on antioxidant functions.

Parameters	Day	Treatments				SEM	*p*-Value
		OS 0	OS 0.20	OS 0.30	OS 0.45		
**GSH-px (U/mg protein)**	36th day	30.02 ^b^	32.608 ^a^	34.08 ^a^	34.218 ^a^	0.516	0.024
	72nd day	32.77 ^c^	35.90 ^b^	38.14 ^a^	38.67 ^a^	0.538	0.011
**SOD (U/mg protein)**	36th day	18.95 ^b^	22.00 ^ab^	23.99 ^ab^	28.40 ^a^	1.669	0.003
	72nd day	24.85 ^b^	28.23 ^b^	30.36 ^b^	36.64 ^a^	1.350	0.001
**DPPH (mmol DPPH/L)**	36th day	38.10 ^b^	46.09 ^ab^	46.09 ^ab^	50.57 ^a^	2.430	0.017
	72nd day	40.83 ^b^	49.82 ^ab^	47.61 ^ab^	52.86 ^a^	2.979	0.012

OS 0, OS 0.20, OS 0.30, and OS 0.45 represent supplementation of 0, 0.20, 0.30, and 0.45 ppm OS in basal diet; SEM: standard error of means. Values labeled with different lowercase letter within a column indicate significant differences between groups (*p* < 0.05).

**Table 9:** Effect of organic selenium in the diet of laying hens on serum minerals.

Parameters	Day	Treatments				SEM	*p* Value
		OS 0	OS 0.20	OS 0.30	OS 0.45		
Ca mg/dL	36th day	6.08	6.81	7.21	9.14	1.064	0.342
	72nd day	7.56	6.36	8.61	9.26	0.933	0.302
P mg/dL	36th day	3.59	4.84	5.61	5.66	0.948	0.454
	72nd day	4.18	5.38	6.09	7.13	0.388	0.243
Mg mg/dL	36th day	1.59	3.21	2.40	2.47	0.508	0.306
	72nd day	2.27	2.45	1.86	3.73	0.351	0.070
K mmol/L	36th day	5.19	4.61	6.17	5.11	0.557	0.349
	72nd day	4.46	5.43	4.83	5.82	1.476	0.910
Cl mmol/L	36th day	113.34	116.75	131.95	111.61	10.558	0.568
	72nd day	116.83	112.65	141.92	117.12	11.061	0.352
Na mmol/L	36th day	148.14	144.53	142.08	150.51	3.061	0.342
	72nd day	151.53	145.55	155.01	151.53	3.418	0.295

OS 0, OS 0.20, OS 0.30, and OS 0.45 represent supplementation of 0, 0.20, 0.30, and 0.45 ppm OS in basal diet; SEM: standard error of means.

**Table 10:** Effect of organic selenium in the diet of laying hens on bone mineralization.

Parameters	Day	Treatments				SEM	*p*-Value
		OS 0	OS 0.20	OS 0.30	OS 0.45		
Ash (%)	36th day	30.70	34.41	36.95	37.90	1.211	0.079
	72nd day	31.30	34.30	38.55	36.7	1.171	0.054
Calcium (%)	36th day	20.40	20.71	21.69	20.73	0.386	0.253
	72nd day	20.48	20.82	21.60	22.40	0.212	0.065
Phosphorus (%)	36th day	7.70	8.21	9.13	9.35	0.316	0.068
	72nd day	8.43	8.75	9.44	9.81	0.177	0.073

OS 0, OS 0.20, OS 0.30, and OS 0.45 represent supplementation of 0, 0.20, 0.30, and 0.45 ppm OS in basal diet; SEM: standard error of mean.

**Table 11:** Effect of Newcastle disease at different time intervals.

Antibody Titers Against ND	Treatments				SEM	*p* Value
	OS 0	OS 0.20	OS 0.30	OS 0.45		
Day 36	5.081 ^d^	6.056 ^c^	6.404 ^b^	6.917 ^a^	0.014	0.022
Day 72	6.488 ^d^	9.201 ^c^	9.549 ^b^	9.829 ^a^	0.020	0.020

OS 0, OS 0.20, OS 0.30, and OS 0.45 represent supplementation of 0, 0.20, 0.30, and 0.45 ppm OS in basal diet; SEM: standard error of means. Values labeled with different lowercase letter within a column indicate significant differences between groups (*p* < 0.05).

## Data Availability

The raw data supporting the conclusions of this article will be made available by the authors upon request.
